# Archetypes of Family Health Climates for Nutrition and Physical Activity Among Families in Singapore: A Cross-Sectional Study

**DOI:** 10.3390/healthcare14050669

**Published:** 2026-03-06

**Authors:** Dhiya Mahirah, Yi-Ching Lynn Ho, Zi Hsuan Chia, Mary Su-Lynn Chew, Xuan Han Koh, Jin-Jin Lim, Julian Thumboo, Kinjal Doshi

**Affiliations:** 1 Centre for Population Health Research and Implementation, Singapore Health Services, Singapore 150167, Singapore; yiching.lynn.ho@gmail.com (Y.-C.L.H.); mary.chew.s.l@singhealth.com.sg (M.S.-L.C.); lim.jin.jin@singhealth.com.sg (J.-J.L.);; 2 Group Finance Analytics, Singapore Health Services, Singapore 150167, Singapore; 3 Health Services Research, Changi General Hospital, Singapore 529889, Singapore; koh.xuan.han@singhealth.com.sg; 4 Department of Rheumatology and Immunology, Singapore General Hospital, Singapore 169608, Singapore; 5 Medicine Academic Clinical Program, Duke-NUS Medical School, Singapore 169857, Singapore; 6 Department of Psychology, National University Singapore, Singapore 119077, Singapore; kinjal@insightconsult.com.sg

**Keywords:** family health climate, Singapore, cluster analysis, archetypes, physical activity, nutrition

## Abstract

**Highlights:**

**What are the main findings?**

- Four family health climate archetypes were identified, reflecting differences in routines, resources and engagement in healthy behaviours
- Socioeconomic and family structure factors shape how families support physical activity and nutrition

**What are the implications of the main findings?**

- Interventions should be tailored to the needs and contexts of different family types.
- Family-based strategies should be supported by the broader community, and policy efforts should support family-based strategies

**Abstract:**

**Background:** Poor lifestyle choices contribute significantly to non-communicable chronic diseases. Given the family’s influence on health behaviours, this study aimed to identify distinct family archetypes based on family health climates for physical activity and nutrition to inform targeted family-based health promotion strategies. **Methods:** Two hundred family dyads (≥15 years old, cohabiting) in Singapore completed a survey assessing family health climates (physical activity and nutrition), together with individual behaviours, and family lifestyle behaviours. Based on family health climate scores, K-means clustering identified family archetypes. Random Forest analysis determined key contributing constructs to the clusters. Chi-square and ANOVA tests compared socio-demographics, family and individual behaviours, and sleep quality across archetypes. **Results:** Four family health climate archetypes were identified: Survivors (16%) had poor health climate scores for both physical activity and nutrition, lower socioeconomic status, infrequent family meals, and poorer diet and sleep quality. Nourished Sedentary (16%) had a poor climate for physical activity but a positive climate for nutrition, higher socioeconomic status, frequent family meals, and limited physical activity. Satisficers (41%) had moderate health climates for both, with average socioeconomic status and engagement in healthy behaviours. Flourishers (27%) had positive health climates for both higher socioeconomic status, frequent family meals, healthy food choices, and greater physical activity engagement. **Conclusions:** The identification of four distinct family health climate archetypes demonstrates that health-related behaviours cluster at the family level rather than solely at the individual level. These findings underscore the importance of targeting family systems in health promotion and support the development of tailored, family-specific strategies.

## 1. Introduction

The global rise in non-communicable chronic diseases presents a pressing public health challenge worldwide, driven in part by unhealthy lifestyle choices such as sedentary behaviour, poor dietary habits, and insufficient sleep [1,2,3,4]. While health promotion efforts have traditionally targeted individuals, there is increasing recognition that focusing solely on individual behaviour change may be insufficient, given the powerful influence of environmental factors, including the family context [5,6,7,8].

The family unit represents a critical social environmental influence in which health behaviours are modelled, reinforced and shaped through everyday interactions [9,10,11]. Consistent with this, behavioural risk factors and chronic disease outcomes have been shown to cluster within families and spouses, suggesting that shared environments, beyond shared genetics, play a significant role [12,13]. One framework that captures these shared influences is the family health climate (FHC), defined as the shared perceptions and cognitions regarding healthy lifestyles within a family unit [14]. The mechanisms include reciprocal modelling of health behaviours, support for health-related practices (e.g., access to nutritious food or exercise equipment), and the development of shared health-related norms [15]. Studies have shown that FHC domains for physical activity (FHC-PA) and nutrition (FHC-NU) are associated with corresponding individual lifestyle behaviours, including physical activity and dietary patterns [14,16].

Beyond documented associations between FHC domains and individual behaviours, less is known about whether families can be meaningfully grouped according to shared family health climate patterns. Prior research demonstrates that health behaviours co-occur at the family level. For example, Niermann et al. [17] identified three family clusters (“healthy”, “unhealthy,” and “divergent”) based on physical activity, nutrition, and screen time, with FHC scores differing significantly across these behaviour-defined groups. Specifically, clusters were derived from individual behavioural indicators, and FHC was merely compared across clusters as a descriptive characteristic. Given that FHC captures shared perceptions, norms and interaction patterns related to health, clustering families directly using FHC domains may offer a complementary way to conceptualise families as holistic health environments [14,15]. Moreover, the use of validated FHC scales may provide a more feasible and scalable approach to identifying family-level health patterns in community settings rather than detailed behavioural assessments.

This approach is particularly relevant in Singapore’s socio-cultural context, where multigenerational co-residence of parents, adult children, and sometimes grandparents remains prevalent [18], creating shared living environments that may shape daily routines and health-related interactions. Intergenerational dynamics within these households may influence dietary norms, communication about nutrition and opportunities for shared physical activity [7,19,20]. In addition, national preventive initiatives, including the War on Diabetes and more recently, HealthierSG and GrowWell SG, emphasise upstream and preventive approaches to health [21,22]. Understanding how family health climates are configured within this context may therefore provide actionable insights for tailoring family-centred interventions.

Therefore, this study aimed to identify distinct family archetypes in Singapore based on patterns of FHC-PA and FHC-NU scores, and to examine their associations with demographic characteristics and lifestyle behaviours, including physical activity, diet and sleep.

## 2. Materials and Methods

The study is based on a cross-sectional survey conducted in Singapore over 12 weeks, from October 2020 to January 2021. It was approved by the institutional ethics committee (CIRB Ref. 2020/2195).

### 2.1. Participants and Procedures

This study analysed data from a cross-sectional online survey involving 200 family dyads (400 individuals) residing in the same household in Singapore. A family dyad was defined as any two cohabiting family members aged 15 years or older living in the same household. Dyads included a range of relationship types (e.g., parent–child, spouses, siblings, and other adult family members), reflecting diverse household compositions rather than a single predefined family role structure.

Participants were recruited through convenience sampling via a commercial survey panel and social media advertisements. Interested individuals were screened for eligibility and invited to participate together with a cohabiting family member who also met the inclusion criteria. Each dyad member completed the survey independently using a personalised survey link. Data quality checks were applied to identify invalid responses, and only complete dyad datasets that met predefined quality criteria were retained for analysis. Recruitment continued until 200 valid dyad responses were obtained.

The target sample size was determined based on prior validation work on the Family Health Climate (Singapore) (FHC (Sg)) scales using the same dataset, which recommended a minimum number of participants per item for scale-based analyses [23].

### 2.2. Measures

Self-reported measures were used in the survey, as outlined below. Data on participants (age, sex, race, education, occupation, marital status, height, weight) and their household characteristics (type of housing, household income, number of household members, who cooked and planned meals, and whether they lived with a domestic worker) were also collected. In Singapore, housing can be broadly categorised as public or private. Public housing refers to government-subsidised apartments managed by the Housing and Development Board (HDB), with smaller units being more affordable. We classified housing types into three groups based on estimated cost: small- to medium-sized public housing (4-room HDB flats or smaller), large public housing (5-room flats and executive HDB flats), and private housing (condominiums and landed properties).

#### 2.2.1. Family Health Climate Scales

The Singapore version of the scales (FHC (Sg)) was used. Based on the original Family Health Climate scales created for the German population, the FHC (Sg) scales have been adapted, validated and translated for the multi-ethnic population in Singapore [23]. The physical activity domain, FHC-PA (Sg), consists of three factors (value, cohesion, and information) with a total of 16 items. The nutrition domain, FHC-NU (Sg), consists of four factors (value, cohesion, communication, and consensus), totalling 17 items. Responses are made on a four-point Likert scale rating of 0 = “strongly disagree”, 1 = “somewhat disagree”, 2 = “somewhat agree”, 3 = “strongly agree”. The ranges of possible scores for FHC-PA (Sg) and FHC-NU (Sg) are from 0 to 48 and 0 to 51, respectively, with higher scores indicating a better family health climate. For simplicity, the acronyms “FHC-PA” and “FHC-NU” will refer to the Singapore version of the scales throughout the article. The FHC scores reflect individual perceptions of family health climate and were analysed jointly at the dyad level, rather than aggregated into a single family-level mean or consensus score.

#### 2.2.2. International Physical Activity Questionnaire (IPAQ)

Physical activity was assessed using the IPAQ, which captures time spent in different domains of activity over the last seven days. The 27-item questionnaire has demonstrated acceptable test–retest reliability and criterion validity across multiple countries [24]. Following the IPAQ scoring protocol, we calculated energy expenditure in metabolic equivalents (MET-min/week) for recreation, sport, and leisure-time activities.

#### 2.2.3. Diet Screener

Dietary intake was evaluated using a 37-item diet screener [25] that measures the frequency of consumption of major food groups over the past year. It includes items covering grains, protein sources, dairy products, fruits, vegetables, and sugar- and fat-rich foods, and beverages. In the Singapore context, the screener has shown reasonable validity and good reproducibility when benchmarked against a detailed Food Frequency Questionnaire [25].

To derive an overall dietary quality indicator, we applied the Dietary Approaches to Stop Hypertension (DASH) scoring index [26]. Seven food categories were included: whole grains, fruits, vegetables, nuts and legumes, low-fat dairy, red processed meats (reversed scoring), and sugar-sweetened beverages (reversed scoring). Sodium was excluded due to the diet screener’s limited ability to accurately estimate sodium intake, given the variability in sodium content across Asian dishes [25]. Total DASH scores ranged from 7 to 35, with higher scores reflecting healthier dietary patterns.

#### 2.2.4. Pittsburgh Sleep Quality Index (PSQI)

We used the 10-item PSQI to assess a person’s sleeping habits in the past month. The PSQI has been tested in Asian contexts as reliable for adults in determining their sleep quality, disturbance, and efficiency [27]. The PSQI scores range from 0 to 21, with higher scores indicating poorer sleep quality.

#### 2.2.5. Family-Related Lifestyle Behaviours

Items assessing family-level health routines and support were included to capture shared behavioural contexts [14]. These items examined the frequency of engaging in physical activities or meals as a family, encouragement among family members to partake in physical activities or healthy eating, and the availability of healthy and unhealthy foods in the household. A five-point Likert scale (1 = “Never” to 5 = “Very often”) was used.

### 2.3. Data Analysis

#### 2.3.1. Data Preparation

Data from all 200 dyads were used. As responses were necessary to proceed with the survey, there was no missing data. However, PSQI scores for six participants were excluded due to implausible sleep durations (e.g., negative hours). For household demographic variables, discrepancies between dyad members were identified in 36 dyads for household income and 3 dyads for housing type. As we could not recontact the anonymised participants, we reconciled the discrepancies by taking the response of the older dyad member, based on the assumption that older household members are more likely to have oversight of household-level characteristics. If both dyad members were the same age, we used the male participant’s response. This reconciliation rule was predefined and applied uniformly across all dyads for household-level demographic variables. Family-level continuous variables (e.g., frequency of family engagement in physical activities or meals together) were aggregated by computing the mean of the two dyad members’ responses.

Household lifecycle stages were categorised using the Singapore Housing and Development Board’s [28] classification system, which delineates households into three distinct stages. Families with dependent children are defined as those in which the eldest child is 20 years old or younger. Families with independent children encompass three sub-categories: households where the eldest unmarried child is 21 years or older, households where the eldest child is married and cohabiting with their parents, or potentially with prior marriage history, and households where the eldest child’s age or marital status is unknown, or where the eldest child is unidentified. Lastly, families without children include married couples with both spouses aged 64 years or younger, married couples with at least one spouse aged 65 years or older, and siblings residing together.

The calculated BMI values were categorised as follows: underweight (BMI < 18.5), healthy (18.5 ≤ BMI < 23), overweight (23 ≤ BMI < 27.5), and obese (BMI ≥ 27.5) [29].

To examine within-family agreement in FHC scores, intraclass correlation coefficients (ICCs) were computed between dyad members using a two-way, random-effects model based on average measures between the dyads to assess absolute agreement [30]. This approach has been described in detail in a prior validation study for the FHC(Sg) scales, where moderate to good agreement between dyad members was observed across FHC-PA and FHC-NU domains [23]. FHC construct scores were calculated at the individual level and were not collapsed into a single dyad-level mean or consensus score. Instead, both dyad members’ FHC construct scores were jointly represented in a dyad-level analytic structure for subsequent clustering.

#### 2.3.2. Cluster Analysis

Family archetypes were identified using a data-driven, unsupervised clustering approach, with no a priori specification of the number or characteristics of clusters. Clustering was performed on dyad-level scores for the 7 FHC constructs (3 FHC-PA constructs and 4 FHC-NU constructs), i.e., a total of 14 dimensions for each dyad. As K-means is a distance-based method, the FHC constructs were standardised using z-scores. Data was arranged and analysed in a row-wise dyadic structure to account for non-independence between dyad members [31]. We used K-Means clustering [32] and determined the optimal number of clusters using two metrics: (a) intra-cluster variation by taking the mean square of the distances of each point within the cluster to all other points in the same cluster, and then calculating the overall mean per cluster. This method is similar to that utilised in the ‘Elbow Method’ [33]; and (b) the silhouette score, which is calculated by taking the mean nearest-cluster distance subtracted by the mean intra-cluster distance. It is then divided by either the former or the latter, whichever is the larger number, giving the silhouette score [34]. Therefore, the ideal number of clusters would result in low intra-cluster variation and a high silhouette score. The number of clusters was selected based on joint consideration of these metrics, with preference given to solutions showing diminishing gains in intra-cluster variation, while maintaining adequate cluster separation and interpretability of the resulting cluster profile.

A secondary Random Forest model was fitted to the data, with the K-Means cluster assignment as the target variable. The model’s feature importance was examined to aid interpretability of the clustering solution by identifying construct(s) that most strongly contributed to cluster differentiation.

The robustness of cluster analysis was ascertained by swapping the order of the domain-construct scores within each dyad. The swapped data was then fed into the trained K-Means clustering model for cluster assignment. Agreement between pre- and post-swap cluster assignments was measured using Cohen’s Kappa statistic, which ranges from −1 to 1 [35]. A high Kappa value indicates strong agreement between the original cluster assignments and the assignments after swapping respondent data, signifying the model’s robustness to the order of participant responses within a dyad. Conversely, a low Kappa score suggests that the order of responses influenced cluster assignments, indicating potential bias.

#### 2.3.3. Comparative Analyses Across Clusters

Inter-cluster variation was calculated for the survey questions by computing the row-wise standard deviation, which describes the degree of variation in responses between clusters for each survey question. Adjusted inter-cluster variation was calculated by dividing the row-wise standard deviation by the row-wise mean. A higher adjusted inter-cluster variation indicates a greater degree of variation in responses between clusters for a particular survey question. Chi-square tests for categorical variables and ANOVA tests for continuous variables were conducted to determine statistically significant differences between the clusters for selected key variables, with *p*-values adjusted using Sidak’s correction.

All data wrangling and modelling were performed using Python (version 3.8.3) (https://python.org) within a Jupyter Notebook environment (version 6.0.3) (https://jupyter.org), which was used to organise, run and document the analyses and generate figures.

## 3. Results

### 3.1. Participant and Household Profile

The sample comprised 200 family dyads residing in the same household. Relationship pairings included parent–child (43%), couples (37.5%), siblings (19%), and one aunt-nephew dyad (0.5%). Most participants lived in public housing (83%) (i.e., HDB flats), with the remainder residing in private condominiums or landed property. Approximately half of households (50.5%) reported a monthly income of $0 to ≤$7500. Participants had a mean age of 42 years (SD = 15.18; range 15 to 85 years old), and 62.7% were female. The sample was predominantly Chinese (86.5%), followed by Malay (6.0%) and Indian (6.3%). Two-thirds had a tertiary education (67.3%). Compared with national population statistics [36], female participants (62.7% vs. 51.1%) and individuals with tertiary education (67.3% vs. 36.1%) were overrepresented in the sample.

### 3.2. Clustering of FHC Scores

**Figure 1:**
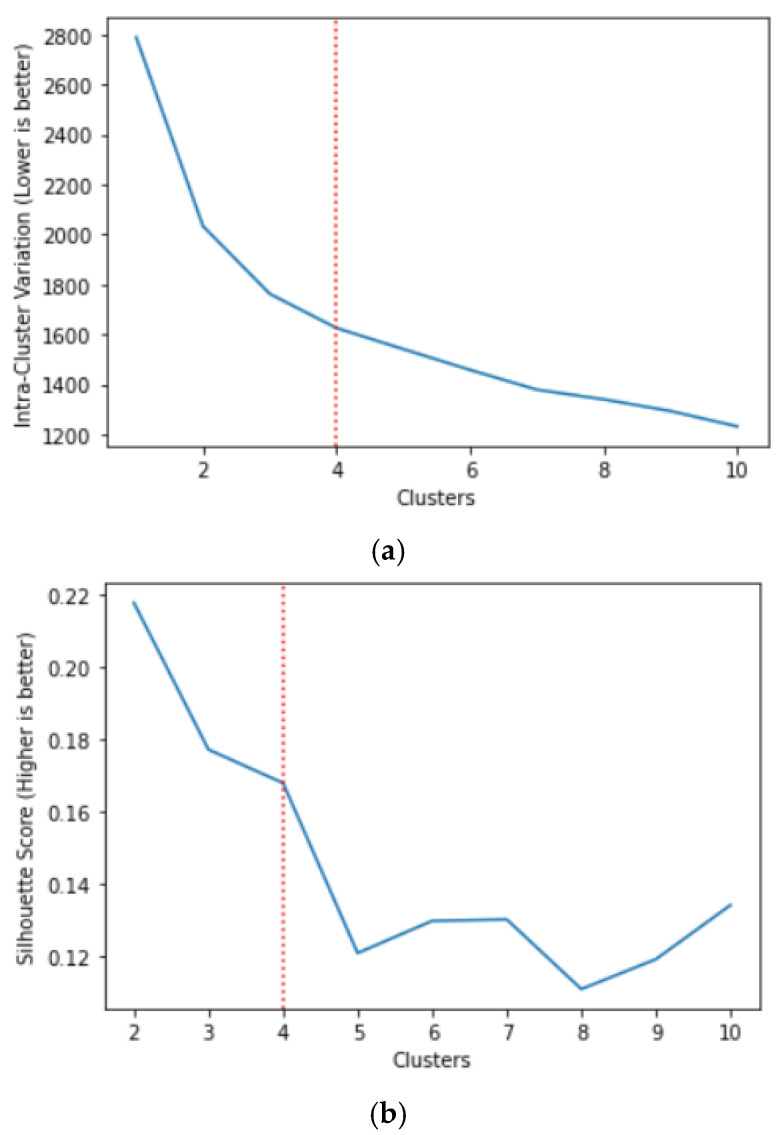
(**a**) Graph of intra-cluster variation against the number of clusters. (**b**) Graph of silhouette scores against the number of clusters.

We identified the optimal number of clusters as 4 by optimising intra-cluster variation (Figure 1a), while ensuring an acceptable silhouette score, for which the higher is better (Figure 1b). This solution represents a balance between within-cluster homogeneity and differentiation between family health climate profiles.

**Figure 2:**
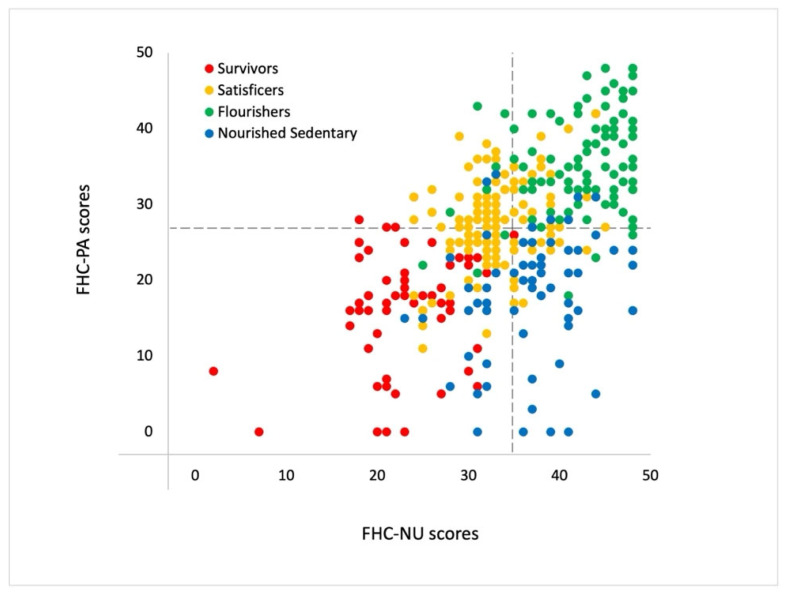
The four archetypes of family health climate scores, with points coloured by cluster assignment. The black dotted lines represent the grand means.

In the four clusters identified (see Table 1, Figure 2), we had a cluster of families with the lowest FHC-PA and FHC-NU scores, comprising 16% of the families, which we termed the Survivors (Cluster 1). The second cluster, termed the Nourished Sedentary, comprised around 17% of families who had poor FHC-PA scores but high FHC-NU scores. Satisficers (Cluster 3) was the largest cluster, representing 40% of families with moderate FHC-PA and FHC-NU scores. The Flourishers accounted for approximately 27% of families and had the highest FHC-PA and FHC-NU scores. Table 2 summarises the key characteristics of each archetype.

To visualise cluster distribution, we calculated the total mean PA and NU scores within each family dyad and plotted them in Figure 2, colouring each family dyad by cluster assignment. Upon visual inspection, we noted that the variation within Satisficers and Flourishers was significantly lower than that of Survivors and Nourished Sedentary. Domain-level patterns for FHC-PA and FHC-NU constructs were consistent with the overall cluster profiles and presented in Supplementary Material Figures S1 and S2.

### 3.3. Domain-Construct Importance to Cluster Assignment

**Figure 3:**
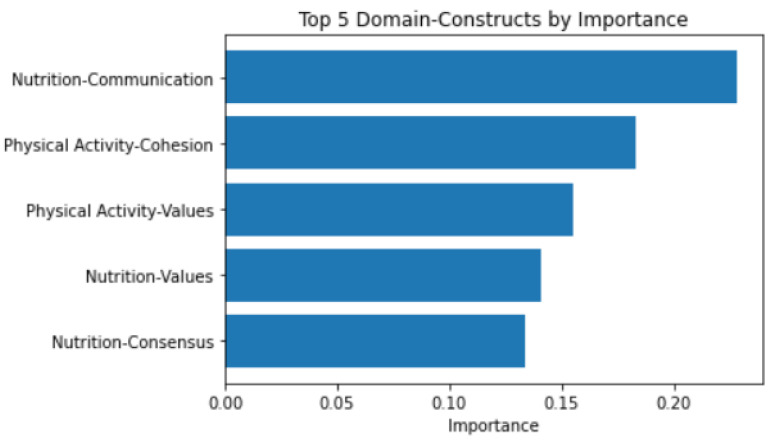
Top 5 domain constructs by importance to cluster assignment.

Figure 3 shows the top five feature importance plots of the secondary Random Forest model. Nutrition communication was the domain construct that most influenced cluster assignment.

### 3.4. FHC Clusters and Demographics

Table 3 shows family demographic variables, including cluster means, the proportion of responses, and chi-square tests. After post hoc Sidak tests, there was no significant difference in the number of household members across clusters. Participants living on private property were more represented by Flourishers than Survivors. On the other hand, participants with a household income of $2501 to $7500 were more highly represented by Satisficers.

Based on the household lifecycle stages categorisation by Singapore’s Housing and Development Board [28], Flourishers had more families living without children than Survivors and Nourished Sedentary. Survivors had no domestic worker planning and cooking their meals compared with the other clusters. Across all clusters, the most common arrangement was for family members to cook and plan meals themselves. Satisficers had a higher proportion of families with domestic workers cooking meals and a lower proportion of families relying on family members for cooking compared to Survivors. Full household and demographic characteristics by family clusters are detailed in Supplementary Materials Table S1.

### 3.5. FHC Clusters with Family Lifestyle Behaviour

Table 4 outlines the cluster means and ANOVA test results for family lifestyle behaviour variables. Clusters with higher FHC scores had higher mean responses for healthy family lifestyle behaviours, while clusters with lower FHC scores had lower mean responses for unhealthy family lifestyle behaviours. Specifically, Flourishers consistently reported mean frequencies close to “regularly” to “very often” for shared physical activity, healthy eating encouragement and availability of healthy foods. In contrast, Survivors reported frequencies below “regularly” across the same 5-point scale. Intermediate patterns were observed among Satisficers and Nourished Sedentary families.

### 3.6. FHC Clusters with Individual Lifestyle Behaviour

Table 5 outlines individual lifestyle behaviour variables across clusters. There was no statistically significant association between BMI categories and family clusters. However, differences were observed for individual physical activity, diet, and sleep outcomes. Specifically, Flourishers reported higher levels of leisure-time physical activity than Survivors and Nourished Sedentary families, with mean weekly activity nearly double that of the lowest-activity cluster (1793 vs. 820 MET-min/week). Differences in diet quality were also evident, with Flourishers scoring higher on the DASH index than Survivors and Satisficers (22.5 vs. 19.0 and 20.1), reflecting healthier dietary patterns. For sleep, Flourishers reported lower PSQI scores than Survivors, corresponding to better overall sleep quality.

Additional regression models adjusting for household income and housing type yielded a similar overall pattern of findings (Supplementary Table S2a,b). Differences between archetypes in shared physical activity, healthy eating encouragement, healthy food availability, DASH scores, and leisure-time physical activity remained statistically significant after SES adjustment.

### 3.7. Clustering Robustness

To analyse the clusters’ robustness, we swapped the order of the dyadic constructs (i.e., member 1 data was swapped with member 2 data). After swapping, 18 changes to cluster assignment were observed, representing 9% of the 200 dyads. The Cohen’s Kappa statistic score was 0.87. The breakdown of the cluster assignment changes can be found in Table S3 (Supplementary Materials). We noted that all changes to cluster assignment occurred between adjacent clusters, i.e., there were no swaps between the extreme clusters of Survivors and Flourishers.

## 4. Discussion

The family environment is a key influence on a family member’s individual and collective health behaviours. Therefore, this study aimed to identify distinct family archetypes based on patterns of family health climate for physical activity and nutrition. A positive family health climate reflects a supportive environment where healthy behaviours related to physical activity and nutrition are encouraged and integrated into family routines, while a negative climate indicates limited family engagement in or support for these behaviours [14]. We identified four distinct clusters corresponding to engagement levels in healthy lifestyle behaviours at both the individual and family levels. Communication about nutrition emerged as the domain that most clearly differentiated clusters, suggesting that relational processes within families play an important role in shaping shared dietary norms.

The Survivors cluster is characterised by negative family health climates in both physical activity and nutrition domains, exhibiting low engagement in healthy lifestyle behaviours at both the family and individual levels. Progressing along the spectrum of engagement, the Nourished Sedentary was marked by a positive family health climate for nutrition, but a negative climate for physical activity. The Satisficers cluster demonstrated moderate scores in both physical activity and nutrition climates. Finally, the Flourishers cluster exhibited high family health climate scores in both domains, reflecting high engagement in family and individual-level health behaviours. The cluster labels are used as descriptive summaries of observed family health climate configurations rather than evaluative categories. Examining each archetype, therefore, provides insight into how these distinct family health climates are expressed within specific contexts.

### 4.1. Survivors

The Survivor cluster, comprising 16% of the sample, had the biggest proportion of families in the lowest household income bracket. Financial constraints in this group may be associated with limited access to resources related to healthy living, such as nutritious food and opportunities for physical activity [37,38]. Several studies have documented associations between lower socioeconomic status and poorer dietary patterns, often linked to food prices and affordability barriers [39]. However, the association between lower socioeconomic status and physical activity is more nuanced, and may vary depending on the domains assessed, such as occupational, transportation and household activities [40,41,42]. Time constraints related to long working hours or caregiving responsibilities may also be relevant considerations when interpreting the lower activity levels observed among survivors.

Financial constraints may further contribute to difficult trade-offs for Survivors, whereby households prioritise basic needs over discretionary health-promoting activities [43]. In lower-income households, expenditures on housing and basic food necessities may take precedence over costs perceived as optional—such as entry fees for recreational facilities, purchasing fresh produce over instant meals, or investing in sports equipment—which may be associated with lower engagement in certain healthy lifestyle behaviours.

Moreover, 97% of Survivor households were multi-generational, with 87.5% including independent adult children residing with parents. While co-residence with adult children may provide practical or emotional support, it may also be associated with increased demands on household resources and time, particularly among socioeconomically disadvantaged families [44,45,46]. Adult children, although independent, may assume caregiving responsibilities for older or younger family members, which may limit time available for recreational physical activity [47]. In Asian cultural contexts, strong norms of filial responsibilities may prioritise family obligations over personal health behaviours, and have been associated with role strain, stress and even physical health issues like sleep disturbances and somatic symptoms [48,49,50,51]. These intersecting socioeconomic and familial factors may help contextualise the lower levels of engagement in recreational activity and poor sleep quality observed in this cluster.

### 4.2. Nourished Sedentary

The Nourished Sedentary cluster, representing 17% of the sample, presented a distinct profile characterised by a positive family health climate for nutrition, but a negative climate for physical activity. This group represented the largest proportion in the highest income bracket (39.4%) with more residents in private housing (18.2% vs. 6.3% for Survivors), suggesting relatively higher socioeconomic status. Additionally, this group included a substantial proportion of multi-generational households (96.8%) and a higher proportion of families with dependent children (21.2% vs. 9.4% for Survivors), indicating potentially greater caregiving responsibilities within these households.

The Nourished Sedentary cluster demonstrated a robust nutrition-related family health climate, reflected in high scores on family-level dietary behaviours, including frequent family meals. Several factors may be associated with the development of structured eating routines in these families, including greater access to nutritious food linked to financial security and the presence of caregivers (e.g., parents and grandparents) who provide nutritional guidance and model healthy eating behaviours during shared meals [20,52,53]. These findings are consistent with prior literature highlighting the family environment as an important context for shared dietary norms [5,14,54]. In Asian contexts, multigenerational households often maintain strong norms around shared meals and traditional dietary practices, which may reinforce structured eating routines and intergenerational transmission of food values [55]. Such cultural patterns may help contextualise the strong nutrition climate observed in this cluster, even in the absence of comparable emphasis on physical activity.

Although the Nourished Sedentary cluster exhibited strong nutrition-related behaviours, this pattern did not extend to physical activity at the family or individual levels. Interestingly, despite less focus on physical activity, this cluster had the highest proportion of individuals within the healthy BMI range (54.6%). However, given the cross-sectional design, it is not possible to determine whether dietary practices, physical activity or other unmeasured factors account for this distribution. Higher socioeconomic status in this cluster may be associated with greater access to healthier, potentially more expensive food options, such as wholegrain rice, which was reported more frequently in this cluster than in the Survivor cluster (refer to Supplementary File, Table S4). Nonetheless, BMI should be interpreted cautiously, as it does not fully capture body composition or physical fitness [56,57]. These findings suggest that interventions aimed at strengthening the family health climate for physical activity may be particularly relevant for this cluster, given the observed imbalance between nutrition and activity domains.

### 4.3. Satisficers

Forty percent of families in the study were within the Satisficers cluster, representing the largest proportion. Satisficers are families that aim for minimally satisfactory outcomes rather than optimal ones [58]. This group demonstrated moderate engagement in both nutrition and physical activity. They had the highest proportion of individuals living in large households (≥6 members), and compared to the Survivors and Flourishers, a higher proportion residing in three-generation households. With higher income levels than the Survivors and the highest proportion of domestic worker employment (17.3%) across the cluster, their relative financial stability and access to household support may be associated with their ability to maintain moderate levels of health-related behaviours alongside lifestyle demands.

The Satisficers reported the highest reliance on domestic workers for meal preparation and the lowest proportion of family members involved in meal planning and cooking. This household arrangement may be consistent with competing obligations in larger multigenerational households. Reliance on external support for meals may prioritise efficiency and convenience, which aligns with their moderate nutrition engagement. Furthermore, the availability of caregiving grants and levy concessions for hiring migrant domestic workers in Singapore may make this arrangement more accessible for middle-income households [59,60].

Access to domestic support may also be associated with how time and responsibilities are distributed within the household, which could relate to the patterns of physical activity observed in this cluster. Although Satisficers did not exhibit the high levels of nutrition and physical activity observed among the Flourishers, they maintained moderate scores across both domains. As these scores approximate the overall sample means, they reflect adequacy rather than optimal health behaviour levels. This cluster may therefore benefit from targeted support that builds upon existing routines to enhance nutritional quality and physical activity.

### 4.4. Flourishers

The Flourisher cluster represents families with the most favourable family health climates and the highest levels of engagement in both physical activity and nutrition. This cluster had the largest proportion residing in private housing (25.9%) and a relatively higher proportion in the highest income bracket (37.0%). Most members lived in single-generation households primarily consisting of couples and siblings. Their relative economic advantage may be associated with greater access to resources such as fitness facilities and a wider range of healthy food options, which may be consistent with their reported engagement in healthy lifestyle behaviours [61,62,63]. For example, they reported the highest frequency of wholegrain food consumption, including red or brown rice, wholegrain noodles and wholemeal bread (refer to Supplementary File Table S4).

While these factors align with favourable health-related patterns, it is important to consider how their unique household structures may present both opportunities and challenges over time. Single-generation households may experience reduced built-in family support compared to multigenerational arrangements, and social disconnectedness has been associated with poorer health outcomes in prior research [64]. However, social support networks outside the household could mitigate such risk [65]. Additionally, greater autonomy over schedules and food choices in smaller households may facilitate the prioritisation of health behaviours that support long-term independence [66]. Recognising the evolving needs of this demographic remains important for sustaining well-being across the lifespan [65,67].

### 4.5. Implications and Future Direction

Beyond standard individual-focused health promotion programmes, identifying family archetypes and their distinct strengths and challenges may inform the design of more contextually tailored health promotion strategies. In the Singapore context, Survivor families may benefit from initiatives addressing socioeconomic barriers and time constraints by expanding access to affordable, healthy food and promoting time-efficient lifestyle strategies. Community-based initiatives like community gardens and healthy food prescriptions have been associated with improved access to nutritious foods [68,69]. Incorporating physical activity into daily routines, such as utilising public transportation or engaging in active chores, may offer realistic entry points for behaviour change [42]. Interventions should prioritise reducing practical barriers and simplifying access to health information and resources through accessible community channels (e.g., mobile-based information dissemination or community networks), especially for resource-constrained households.

Nourished Sedentary families, with strong dietary patterns and potentially strong family values around nutrition, may benefit from interventions that build on these strengths to promote engagement in physical activity. For example, leveraging established meal routines as touchpoints for goal-setting or family-based activity planning may help extend positive nutrition climates into the physical activity domain. Satisficers may benefit from structured reinforcement strategies that support the maintenance and gradual enhancement of existing healthy routines. Structured goal-setting, periodic feedback, and family-based planning approaches may help sustain engagement without requiring substantial additional resources.

Broader systemic factors influencing family health behaviours must also be considered [70]. Interventions that support families in achieving moderate, sustainable improvements—rather than exclusively targeting optimal health climates—may represent a pragmatic short-term approach. The adaptive strategies observed among Satisficers may offer transferable insights for other archetypes. At the same time, examining the enabling conditions of Flourishers may inform upstream policy development. Future qualitative research could explore the relational and communicative processes that distinguish these clusters within their socioenvironmental context.

It is also important to interpret these archetypes in light of the sample composition. The dyads comprised parent–child (43%), couple (37.5%), and sibling (19%) pairs, with a mean age of 42 years (range 15–85 years), reflecting representation across adolescent, adult, and older adult life stages. The identified family health climate patterns, therefore, capture a mixture of adolescent-adult and adult-adult household dynamics. Intervention strategies may need to account for developmental stage and relational context within each archetype. For example, approaches for parent-adolescent households may differ from those suited to couple-based or multigenerational adult households. Furthermore, given the prominence of communication-related domains in differentiating archetypes, interventions may benefit from incorporating relational components—such as family-based health coaching, guided goal-setting conversation, or structured dialogue around nutrition practices—in addition to resource provision and individual behaviour change strategies [15].

A common theme across all archetypes is the presence of resource limitations—financial constraints, time scarcity, and limited energy—that may be associated with variations in families’ ability to engage in healthy behaviours. This highlights the importance of addressing both individual behaviours and broader social determinants of health. Future research should prioritise the development and evaluation of multi-level interventions that combine individual- and family-level support with community-based initiatives and policy efforts to address systemic health inequities [70,71]. While providing practical strategies for optimising existing resources is important, policy approaches aimed at reducing financial barriers to healthy food, improving time flexibility (e.g., paid family leave), and creating supportive environments (e.g., accessible parks) may further contribute to a more equitable approach to family health promotion [72,73].

### 4.6. Limitations

The study relied on dyadic responses within each household to measure family-level factors. While this approach is appropriate for two-person families, in larger families, the assumption is that dyadic responses reflect the experiences and perceptions of the broader family unit, which may not always be the case. Discrepancies between dyad members’ responses on household-level demographic variables were resolved using a predefined rule prioritising the older or male respondent. Although applied consistently, this approach may reflect implicit assumptions about family roles and decision-making. The study also did not account for the length of time dyad members had co-resided, implicitly assuming an extended shared living history.

All measures were self-reported and collected via an online questionnaire, which may be subject to recall and social desirability bias. As participants were recruited through convenience sampling (via an online survey panel and social media outreach), the sample may be subject to sampling bias. Individuals with lower digital access or literacy may have been less likely to participate, and women and those with higher educational attainment were overrepresented relative to national distributions. There were also relatively few participants living in small public housing flats (n = 4), and ethnic minority and socioeconomically disadvantaged households were underrepresented. These factors should be considered when interpreting the applicability of the identified archetypes and their generalisability to the broader Singapore population. As with all cross-sectional studies, causal inferences cannot be made.

## 5. Conclusions

This study identified four distinct archetypes of family health climates, profiled by demographics and lifestyle behaviours. Flourisher families demonstrated the highest levels of engagement in physical activity and nutrition, while Survivor families showed the lowest levels, with intermediate patterns observed among Nourished Sedentary and Satisficer clusters. These findings provide insight into how families, within their unique socioenvironmental contexts, exhibit different configurations of health-related practices. They highlight the interconnected nature of family health and emphasise the value of examining family systems rather than individual behaviours in isolation. Differences between archetypes were observed alongside variations in socioeconomic factors, family routines and shared health-related values and practices. Future studies would benefit from examining which subdomains of the family health climate (e.g., value, communication, or cohesion) are most strongly associated with healthy lifestyle behaviours. Identifying the family characteristics and supportive processes associated with more favourable family health climates, particularly among Flourishers and to some extent, Satisficers, may inform the development of preventive family-based interventions tailored to households resembling the Survivors and Nourished Sedentary archetypes.

## Figures and Tables

**Table 1:** Clusters and their corresponding mean total Physical Activity (PA) and Nutrition (NU) scores and number of family dyads within.

Cluster	Name of Cluster	Mean FHC-PA Score	Mean FHC-NU Score	Number of Dyads
1	Survivors	17.4	23.9	32
2	Nourished Sedentary	17.8	36.8	33
3	Satisficers	28.5	33.1	81
4	Flourishers	35.6	42.6	54

**Table 2:** Summary of family health climate archetypes.

Cluster	Proportion of Dyads	Family Health Climate Profile	Sociodemographics	Family Routines and Health Behaviours
Survivors	16% (n = 32)	Lowest FHC-PA and FHC-NU scores	Lower-income profile; predominantly 2-generation public housing households with independent adult children	Infrequent shared physical activity and family meals; lower availability of healthy foods
Nourished Sedentary	17% (n = 33)	Low FHC-PA, high FHC-NU	Mixed-to-higher income profile; higher proportion of multigenerational households with dependent children	Frequent family meals and healthy eating practices, but low engagement in physical activity
Satisficers	40% (n = 81)	Moderate FHC-PA and FHC-NU	Middle-income profile; predominantly 2-generation public housing households with independent adult children	Moderate engagement in both healthy eating and physical activity routines, and greater use of domestic workers for meal preparation
Flourishers	27% (n = 54)	Highest FHC-PA and FHC-NU scores	Higher income profile; greater private housing representation; higher proportion of single-generation households	Frequent shared physical activity and family meals, high availability of healthy foods

**Table 3:** Household factors within each cluster.

Variables	Survivors	Nourished Sedentary	Satisficers	Flourishers	X^2^ Tests and Post Hoc Comparisons
No. of generations in household					
1 generation	3.13% (n = 2)	3.03% (n = 2)	12.4% (n = 20)	20.4% ** (n = 22)	X^2^ = 23.4, *p* = 0.001 Survivors-2 generations (adjusted *p* = 0.002) Flourishers-1 generation (adjusted *p* = 0.0007)
2 generations	87.5% ** (n = 56)	72.7% (n = 48)	69.1% (n = 112)	64.8% (n = 70)	
3 generations	9.38% (n = 6)	24.2% (n = 16)	18.5% (n = 30)	14.8% (n = 16)	
Housing					
Small-medium public housing flats	56.3% (n = 36)	48.5% (n = 32)	56.8% (n = 92)	40.7% (n = 44)	X^2^ = 15.0, *p* = 0.02 Flourishers-Private Housing (adjusted *p* = 0.00385)
Large public housing flats	37.5% (n = 24)	33.3% (n = 22)	28.4% (n = 46)	33.3% (n = 36)	
Private housing	6.25% (n = 4)	18.2% (n = 12)	14.8% (n = 24)	25.9% ** (n = 28)	
Household Income					
$0–$2500	15.6% (n = 10)	9.09% (n = 6)	9.88% (n = 16)	9.26% (n = 10)	X^2^ = 21.1, *p* = 0.012 Satisficers-$2500–$7500 (adjusted *p* = 0.00034) Satisficers-Above $10,001 (adjusted *p* = 0.00137)
$2501–$7500	37.5% (n = 24)	27.3% (n = 18)	50.6% ** (n = 82)	33.3% (n = 36)	
$7501–$10,000	21.9% (n = 14)	24.2% (n = 16)	19.8% (n = 32)	20.4% (n = 22)	
Above $10,001	25.0% (n = 16)	39.4% (n = 26)	19.8% ** (n = 32)	37.0% (n = 40)	
Household Lifecycle Stages					
Household with dependent children	9.38% (n = 6)	21.2% (n = 14)	13.6% (n = 22)	11.1% (n = 12)	X^2^ = 24.6, *p* < 0.001 Flourishers-Family without children (adjusted *p* = 0.00013)
Household with independent (adult) children	87.5% (n = 56)	75.8% (n = 50)	74.1% (n = 120)	66.7% (n = 72)	
Household without children	3.13% (n = 2)	3.03% (n = 2)	12.4% (n = 20)	22.2% ** (n = 24)	
Employment of a domestic worker					
Yes	0.00% ** (n = 0)	9.09% (n = 6)	17.3% ** (n = 28)	9.26% (n = 10)	X^2^ = 15.0, *p* = 0.002 Survivors-No (adjusted *p* = 0.00214) Satisficers-Yes (adjusted *p* = 0.00093)
The person who cooks in the household					
No one	6.25% (n = 4)	3.03% (n = 2)	8.64% (n = 14)	5.56% (n = 6)	X^2^ = 15.8, *p* = 0.015 Satisficers-Only DW cooks (adjusted *p* = 0.00261) Satisficers-Family member (adjusted *p* = 0.00084)
Domestic worker (DW)	0.00% (n = 0)	9.09% (n = 6)	13.6% ** (n = 22)	5.56% (n = 6)	
Family member	93.8% (n = 60)	87.9% (n = 58)	77.8% ** (n = 126)	88.9% (n = 96)	

** *p* < 0.01.

**Table 4:** Mean responses by cluster to family lifestyle behaviour questions.

Variables	Survivors	Nourished Sedentary	Satisficers	Flourishers	ANOVAs and Sidak Post Hoc Comparisons
On a Likert scale of 1 being ‘never’, 3 being ‘regularly’ and 5 being ‘very often’:					
How often does your family engage in physical activities together?	1.72	1.77	2.37	3.08	F(3,396) = 53.1, *p* < 0.001 All post hoc pairwise comparisons sig. (*p* < 0.001) except Survivors-Nourished Sedentary
How often do family members encourage each other to engage in physical activities?	2.22	2.18	2.80	3.52	F(3,396) = 37.7, *p* < 0.001 All post hoc pairwise comparisons sig. (*p* < 0.001) except Survivors-Nourished Sedentary
How often does your family have meals together each week?	3.00	3.86	3.57	4.05	F(3,396) = 11.3, *p* < 0.001 Post hoc comparisons: Survivors-Satisficers (*p* = 0.008) Survivors-Flourishers (*p* < 0.001) Survivors-Nourished Sedentary (*p* < 0.001) Satisficers-Flourishers (*p* = 0.008)
How often do family members encourage each other to eat healthily?	2.38	3.76	3.21	4.04	F(3,396) = 44.7, *p* < 0.001 Post-hoc comparisons: Survivors-Satisficers (*p* < 0.001) Survivors-Flourishers (*p* < 0.001) Survivors-Nourished Sedentary (*p* < 0.001) Satisficers-Flourishers (*p* < 0.001) Satisficers-Nourished Sedentary (*p* = 0.001)
How often are healthy foods (e.g., fruits and vegetables) available in the household?	3.23	4.44	3.64	4.31	F(3,396) = 28.2, *p* < 0.001 Post-hoc comparisons: Survivors-Satisficers (*p* = 0.024) Survivors-Flourishers (*p* < 0.001) Survivors-Nourished Sedentary (*p* < 0.001) Satisficers-Flourishers (*p* < 0.001) Satisficers-Nourished Sedentary (*p* < 0.001)
How often are unhealthy foods (e.g., soft drinks, fried snacks) available in the household?	3.08	2.79	2.90	2.36	F(3,396) = 8.27, *p* < 0.001 Post-hoc comparisons: Survivors-Flourishers (*p* < 0.001) Satisficers-Flourishers (*p* < 0.001)

**Table 5:** Individual BMI, physical activity, nutrition, and sleep quality by cluster.

Variables	Survivors	Nourished Sedentary	Satisficers	Flourishers	X^2^ Tests/ANOVAs and Post Hoc Comparisons
BMI					
Underweight	7.81% (n = 5)	13.6% (n = 9)	9.26% (n = 15)	10.2% (n = 11)	X^2^ = 5.91, *p* > 0.05
Healthy	48.4% (n = 31)	54.6% (n = 36)	45.1% (n = 73)	45.4% (n = 49)	
Overweight	26.6% (n = 17)	21.2% (n = 14)	32.1% (n = 52)	32.4% (n = 35)	
Obese	17.2% (n = 11)	10.6% (n = 7)	13.6% (n = 22)	12.0% (n = 13)	
MET-min/week Physical activity during leisure (IPAQ)	900	820	1058	1793	F(3,396) = 3.73, *p* = 0.0115; Post-hoc comparisons: Flourishers-Nourished Sedentary (*p* = 0.035)
Nutrition quality (DASH score)	19.0	21.4	20.1	22.5	F(3,396) = 9.44, *p* < 0.001; Post-hoc comparisons: Survivors-Flourishers (*p* < 0.001) Satisficers-Flourishers (*p* < 0.001) Survivors-Nourished Sedentary (*p* = 0.025)
Sleep quality (PSQI score)	6.9	5.7	5.7	5.1	F(3,396) = 3.29, *p* = 0.0207; Post hoc comparisons: Survivors-Flourishers (*p* = 0.011)

## Data Availability

The datasets presented in this study are not publicly available due to the stipulations from the local institutional review committee on data sharing beyond the approved study members. Request to access the dataset should be directed to: shq_dymm@nus.edu.sg.

## Supplementary Materials

The following supporting information can be downloaded at: https://www.mdpi.com/article/10.3390/healthcare14050669/s1. Figure S1: Mean FHC-PA z-scores by clusters. Error bars represent standard errors. Figure S2: Mean FHC-NU z-scores by clusters. Error bars represent standard errors. Table S1: Full household and demographic characteristics by family clusters. Table S2: (a): SES-adjusted associations between family clusters and family-level lifestyle behaviours. (b): SES-adjusted associations between family clusters and individuals lifestyle behaviours. Table S3: Type and number of changes to cluster assignment. Table S4: Mean responses by cluster to Diet Screener Questionnaire wholegrain food items.

## Abbreviations

The following abbreviations are used in this manuscript:

ANOVA	Analysis of Variance
BMI	Body Mass Index
CI	Confidence Interval
DASH	Dietary Approaches to Stop Hypertension
FHC	Family Health Climate
HDB	Housing and Development Board
ICC	Intraclass Correlation Coefficient
IPAQ	International Physical Activity Questionnaire
MET	Metabolic Equivalent of Task
NU	Nutrition
PA	Physical Activity
PSQI	Pittsburgh Sleep Quality Index
