# Supplementary material for: Archetypes of Family Health Climates for Nutrition and Physical Activity Among Families in Singapore: A Cross-Sectional Study
Source: Healthcare (Basel). 2026 Mar 6;14(5):669. doi: 10.3390/healthcare14050669 (PMC12985157; doi:10.3390/healthcare14050669)
Supplement: Supplementary file 1 [file healthcare-14-00669-s001.zip › healthcare-4097256-supplementary.pdf]

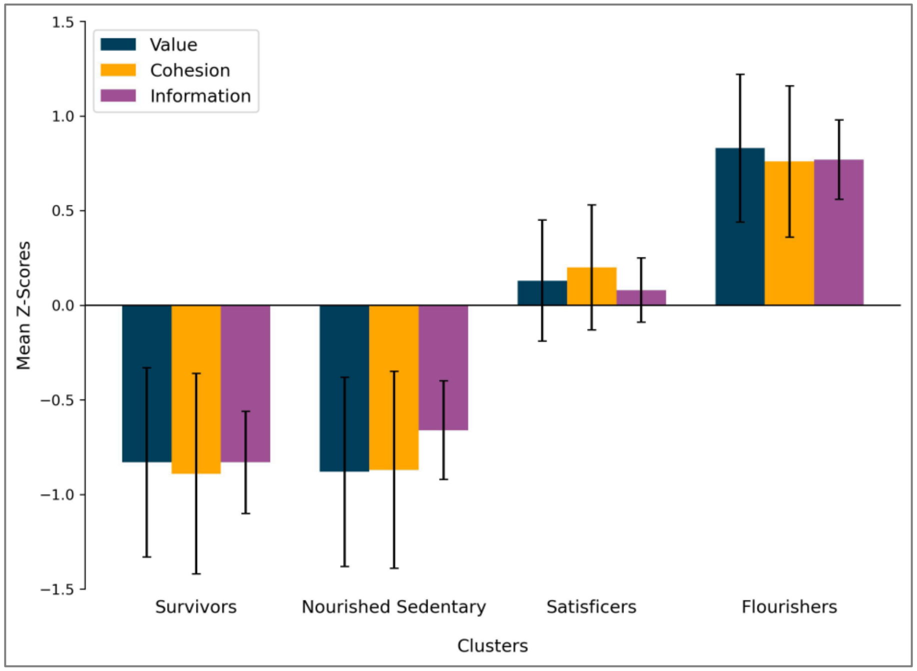


**Figure S1**: **Mean FHC-PA z-scores by clusters. Error bars represent standard errors.**

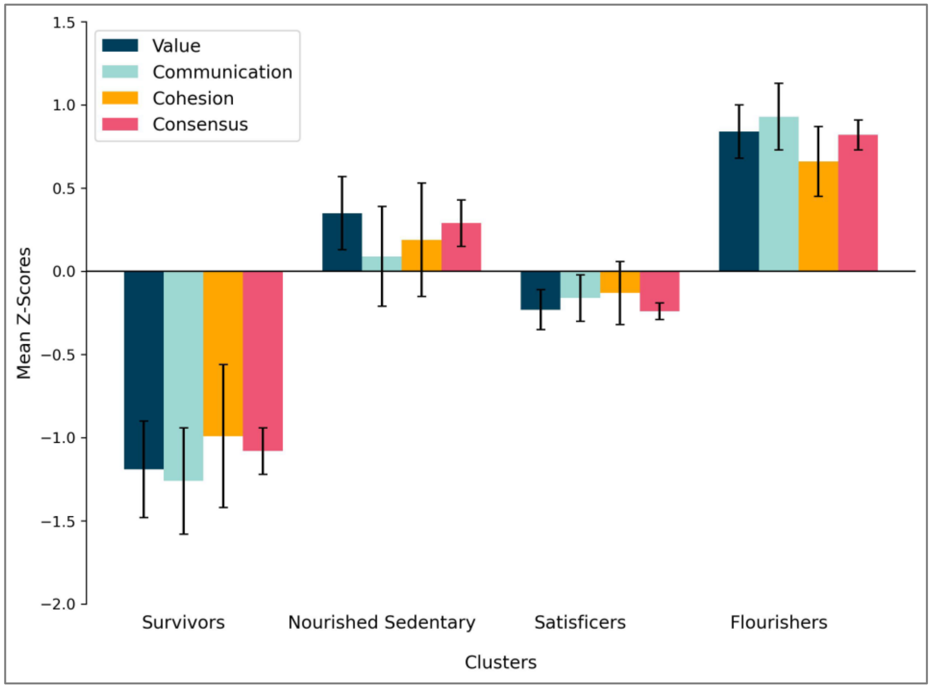


**Figure S2: Mean FHC-NU z-scores by clusters. Error bars represent standard errors.**

**Table S1: Full household and demographic characteristics by family clusters**

| Variables | Survivors | Nourished Sedentary | Satisficers | Flourishers | $X^2$ tests and post-hoc comparisons |
|---|---|---|---|---|---|
| **Household sizes** | | | | | |
| 2-3 members | 50.0% (n=32, z-score=1.06) | 33.3% (n=22, z-score=-1.91) | 40.7% (n=66, z-score=-1.08) | 51.9% (n=56, z-score=1.92) | $X^2$=15.1, p=.019<br><br>No adjusted significant comparisons |
| 4-5 members | 50.0% (n=32, z-score=0.52) | 57.6% (n=38, z-score=1.88) | 46.9% (n=76, z-score=-.03) | 38.9% (n=42, z-score=-1.98) | |
| ≥6 members | 0.00% (n=0, z-score=-2.75) | 9.09% (n=6, z-score=0.03) | 12.3% (n=20, z-score=1.93) | 9.26% (n=10, z-score=0.11) | |
| **No. of generations in household** | | | | | |
| 1 generation | 3.13% (n=2, z-score=-2.29) | 3.03% (n=2, z-score=-2.36) | 12.4% (n=20, z-score=0.44) | 20.4% (n=22, z-score=3.38**) | $X^2$=23.4, p=.001<br><br>Survivors-2 generations (adjusted p=0.002)<br>Flourishers-1 generation (adjusted p=0.0007) |
| 2 generations | 87.5% (n=56, z-score=3.09**) | 72.7% (n=48, z-score=0.24) | 69.1% (n=112, z-score=-0.86) | 64.8% (n=70, z-score=-1.80) | |
| 3 generations | 9.38% (n=6, z-score=-1.77) | 24.2% (n=16, z-score=1.71) | 18.5% (n=30, z-score=0.67) | 14.8% (n=16, z-score=-0.71) | |
| **Housing** | | | | | |
| Small-medium public housing flats | 56.3% (n=36, z-score=0.92) | 48.5% (n=32, z-score=-.45) | 56.8% (n=92, z-score=1.91) | 40.7% (n=44, z-score=-2.50) | $X^2$=15.0, p=.02<br><br>Flourishers-Private Housing (adjusted p=0.00385) |
| Large public housing flats | 37.5% (n=24, z-score=1.03) | 33.3% (n=22, z-score=0.25) | 28.4% (n=46, z-score=-1.28) | 33.3% (n=36, z-score=0.35) | |
| Private housing | 6.25% (n=4, z-score=-2.50) | 18.2% (n=12, z-score=0.28) | 14.8% (n=24, z-score=-0.96) | 25.9% (n=28, z-score=2.89**) | |

| | | | | | |
|---|---|---|---|---|---|
| **Household Income** | | | | | |
| $0 – $2,500 | 15.6% (n=10, z-score=1.46) | 9.09% (n=6, z-score=-0.41) | 9.88% (n=16, z-score=-0.34) | 9.26% (n=10, z-score=-0.49) | $X^2$=21.1, p=.012<br><br>Satisficers-$2,500-$7,500 (adjusted p=0.00034)<br>Satisficers-Above $10,001 (adjusted p=0.00137) |
| $2,501 – $7,500 | 37.5% (n=24, z-score=-0.45) | 27.3% (n=18, z-score=-2.31) | 50.6% (n=82, z-score=3.58**) | 33.3% (n=36, z-score=-1.66) | |
| $7,501 – $10,000 | 21.9% (n=14, z-score=0.19) | 24.2% (n=16, z-score=0.71) | 19.8% (n=32, z-score=-0.51) | 20.4% (n=22, z-score=-0.19) | |
| Above $10,001 | 25.0% (n=16, z-score=-0.68) | 39.4% (n=26, z-score=2.15) | 19.8% (n=32, z-score=-3.20**) | 37.0% (n=40, z-score=2.30) | |
| **Household Lifecycle Stages** | | | | | |
| Household with dependent children | 9.38% (n=6, z-score=-1.05) | 21.2% (n=14, z-score=2.01) | 13.6% (n=22, z-score=0.04) | 11.1% (n=12, z-score=-0.85) | $X^2$=24.6, p<.001<br><br>Flourishers-Family without children (adjusted p=0.00013) |
| Household with independent (adult) children | 87.5% (n=56, z-score=2.60) | 75.8% (n=50, z-score=0.26) | 74.1% (n=120, z-score=-0.16) | 66.7% (n=72, z-score=-2.19) | |
| Household without children | 3.13% (n=2, z-score=-2.38) | 3.03% (n=2, z-score=-2.45) | 12.4% (n=20, z-score=0.18) | 22.2% (n=24, z-score=3.83**) | |
| **Employment of domestic worker** | | | | | |
| Yes | 0.00% (n=0, z-score=-3.07**) | 9.09% (n=6, z-score=-0.54) | 17.3% (n=28, z-score=3.31**) | 9.26% (n=10, z-score=-0.68) | $X^2$=15.0, p=.002<br><br>Survivors-No (adjusted p=0.00214)<br>Satisficers-Yes (adjusted p=0.00093) |
| **Person who plans the meals in the household** | | | | | |
| No one | 9.38% | 6.06% | 12.3% | 3.70% | $X^2$=9.29, p>.05 |

| | | | | | |
|---|---|---|---|---|---|
| | (n=6, z-score=0.27) | (n=4, z-score=-0.78) | (n=20, z-score=2.28) | (n=4, z-score=-2.09) | |
| Domestic worker | 0.00% (n=0, z-score=-1.54) | 3.03% (n=2, z-score=0.02) | 3.70% (n=6, z-score=0.68) | 3.70% (n=4, z-score=0.50) | |
| Family member | 90.6% (n=58, z-score=0.58) | 90.9% (n=60, z-score=0.67) | 84.0% (n=136, z-score=-2.35) | 92.6% (n=100, z-score=1.56) | |
| **Person who cooks in the household** | | | | | |
| No one | 6.25% (n=4, z-score=-0.09) | 3.03% (n=2, z-score=-1.25) | 8.64% (n=14, z-score=1.43) | 5.56% (n=6, z-score=-0.47) | $X^2$=15.8, p=.015 |
| Domestic worker (DW) | 0.00% (n=0, z-score=-2.66) | 9.09% (n=6, z-score=0.19) | 13.6% (n=22, z-score=3.01**) | 5.56% (n=6, z-score=-1.28) | Satisficers-Only DW cooks (adjusted p=0.00261) |
| Family member | 93.8% (n=60, z-score=2.14) | 87.9% (n=58, z-score=0.72) | 77.8% (n=126, z-score=-3.34**) | 88.9% (n=96, z-score=1.32) | Satisficers-Family member (adjusted p=0.00084) |

**p<.05, **p<.01.*

**Table S2a: SES-adjusted associations between family clusters and family-level lifestyle behaviours**

| Outcome | Satisficers vs Survivors β (95% CI), p | Flourishers vs Survivors β (95% CI), p | Nourished sedentary vs Survivors β (95% CI), p |
|---|---|---|---|
| How often does your family engage in physical activities together? | 0.650 (0.390, 0.910), p<.001 | 1.285 (0.966, 1.603), p<.001 | 0.007 (-0.242, 0.256), p=.955 |
| How often does your family have meals together each week? | 0.582 (0.074, 1.090), p=.025 | 0.896 (0.376, 1.416), p=.001 | 0.750 (0.159, 1.342), p=.013 |
| How often do family members encourage each other to engage in physical activities? | 0.556 (0.249, 0.864), p<.001 | 1.182 (0.822, 1.542), p<.001 | -0.076 (-0.446, 0.293), p=.685 |
| How often do family members encourage each other to eat healthily? | 0.841 (0.514, 1.169), p<.001 | 1.604 (1.230, 1.977), p<.001 | 1.358 (0.978, 1.739), p<.001 |
| How often are healthy foods (e.g. fruits and vegetables) available in the household? | 0.412 (0.066, 0.758), p=.020 | 0.974 (0.620, 1.327), p<.001 | 1.095 (0.722, 1.467), p<.001 |
| How often are unhealthy foods (e.g. soft drinks, fried snacks) available in the household? | -0.191 (-0.571, 0.189), p=.324 | -0.709 (-1.085, -0.332), p<.001 | -0.276 (-0.727, 0.175), p=.231 |

*β coefficients from OLS regression models with cluster-robust standard errors. Models adjusted for household income and housing type. Survivors = reference group.*

**Table S2b: SES-adjusted associations between family clusters and individuals lifestyle behaviours**

| Outcome | Satisficers vs Survivors β (95% CI), p | Flourishers vs Survivor β (95% CI), p | Nourished sedentary vs Survivors β (95% CI), p |
|---|---|---|---|
| Nutrition quality (DASH score) | 1.247 (-0.080, 2.574), p=.065 | 3.342 (1.663, 5.021), p<.001 | 2.160 (0.443, 3.877), p=.014 |
| MET-min/week Physical activity during leisure (IPAQ) | 104.7 (-301.6, 511.0), p=.614 | 911.9 (55.9, 1768.0), p=.037 | -88.2 (-521.2, 344.7), p=.690 |

*β coefficients from OLS regression models with cluster-robust standard errors. Models adjusted for household income and housing type. Survivors = reference group.*

**Table S3:** Type and number of changes to cluster assignment.

| Changes to cluster assignment | Number of swaps |
|---|---|
| Between Survivors and Satisficers | 5 |
| Between Satisficers and Flourishers | 7 |
| Between Satisficers and Nourished Sedentary | 6 |

Table S4: Mean responses by cluster to Diet Screener Questionnaire wholegrain food items

| Variables | Survivors | Nourished Sedentary | Satisficers | Flourishers | p-value and post-hoc |
|---|---|---|---|---|---|
| **On a likert scale of 1 – 10, where 1 = "Never or rarely" and 10="6+a day"<br>How often do you consume one serving of:** | | | | | |
| 1. Red or brown rice (including porridge) | 3.12 | 3.89 | 3.43 | 4.08 | $\chi^2(3) = 10.94$, p=0.012, Post-hoc: Survivors – Flourishers (p=0.024) |
| 2. Wholemeal noodles/pasta e.g. brown rice beehoon, wholemeal pasta | 2.70 | 2.58 | 2.86 | 3.22 | $\chi^2(3) = 5.96$, p>.05 |
| 3. Wholemeal bread (including chapati) | 4.12 | 4.23 | 3.99 | 4.94 | $\chi^2(3) = 14.42$, $p$ = 0.002, post-hoc: Satisficer – flourisher (p= 0.002) |

*P-value from Kruskal-Wallis rank sum test and post-hoc pairwise comparison using Wilcoxon rank sum test with Bonferroni correction
